# Serum *miR‐92a‐1* is a novel diagnostic biomarker for colorectal cancer

**DOI:** 10.1111/jcmm.15282

**Published:** 2020-06-20

**Authors:** Ying Shi, Zhibao Liu

**Affiliations:** ^1^ Department of Oncology Cangzhou Central Hospital Cangzhou Hebei China

**Keywords:** Colorectal cancer, Diagnosis, *MiR‐92a‐1*

## Abstract

Colorectal cancer (CRC) is one of the most common cancers worldwide, with high mortality. Abnormally expressed microRNAs (miRNAs) are considered novel biomarkers in cancer diagnosis. The aim of this study was to investigate the diagnostic value of *miR‐92a‐1* in patients with CRC. Serum samples were collected from 148 patients pathologically diagnosed with CRC and 68 gender‐ and age‐matched healthy volunteers. Quantitative real‐time polymerase chain reaction (qRT‐PCR) was used to measure serum *miR‐92a‐1* level. Relationship between *miR‐92a‐1* and clinicopathological features of CRC cases was analysed via chi‐square test. Receiver operating characteristic (ROC) curve was plotted to estimate the diagnostic value of *miR‐92a‐1* in CRC. Serum *miR‐92a‐1* was significantly up‐regulated in CRC patients compared with healthy individuals (*P* < .001). Moreover, *miR‐92a‐1* expression was correlated with TNM stage (*P* = .02), histological stage (*P* = .003), lymph node metastasis (*P* = .003) and distant metastasis (*P* < .001). ROC analysis showed that the area under the ROC curve (AUC) was 0.914, suggesting high diagnostic accuracy of *miR‐92a‐1* in ROC. The optimal cut‐off value was 1.485, with a sensitivity of 81.8% and a specificity of 95.6%. *MiR‐92a‐1* is increased in CRC patients and correlated with aggressive clinical characteristics. Serum *miR‐92a‐1* may be a potential diagnostic biomarker for CRC.

## 1 INTRODUCTION

Colorectal cancer (CRC) is one of the most common malignancies in the world, with high incidence and mortality.^1^ In China, the morbidity and mortality of CRC show rising trends, due to changes in lifestyle and diets.^2^, ^3^ Although treatment methods for CRC have been improved, the survival rates of CRC patients are still not ideal.^4^, ^5^ Tumour stage at diagnosis is a major factor influencing therapeutic effects.^6^ Reportedly, CRC patients diagnosed at early stage were more likely to be cured, while those in advanced stage frequently faced dismal prognosis.^7^ Thus, finding novel and effective biomarkers for early diagnosis of CRC is a valuable way to improve clinical outcome of the patients.

Growing evidences have indicated that the expression patterns of microRNAs (miRNAs) possess close association with tumour development and progression, which might hold the potential to serve as biomarkers for diverse cancers, including CRC.^8^, ^9^, ^10^ MiRNAs are a class of small non‐coding RNAs and play important roles in regulating gene expression.^11^ MiRNAs can bind to the 3′ untranslated region (3′ UTR) of their target mRNAs, thus preventing gene translation.^12^ Aberrant expression of miRNAs can contribute to various human diseases, like malignancy.^13^ In previous tumour investigations, numerous miRNAs were reported to act as oncogenes or tumour suppressors in the progression of CRC. For example, microRNA‐429 (*miR‐429*), *miR‐1260b* and *miR‐10b* played oncogenic roles in CRC development,^14^, ^15^, ^16^ while *miR‐526‐3p*, *miR‐185* and *miR‐452* could inhibit aggressive progression of the malignancy.^17^, ^18^, ^19^ Abnormally expressed miRNAs may provide a novel way for CRC diagnosis.

MicroRNA‐92a‐1 (*MiR‐92a‐1*) is a member of *miR‐17‐92* cluster. *MiR‐17‐92* cluster includes four families: *miR‐17*, *miR‐18*, *miR‐19* and *miR‐92a*.^20^ *MiR‐92a* family includes four members: *miR‐25*, *miR‐92a‐1*, *miR‐92a‐2* and *miR‐363*. The members of *miR‐92a* family play significant roles in the development and progression of CRC, and might serve as biomarkers for the cancer. A study carried out by Li et al demonstrated that the expression of *miR‐25* was up‐regulated in CRC tissues and that its overexpression predicted poor prognosis for the patients.^2^ In the study of Xu et al, the expression profile of *miR‐363* was confirmed to be a novel diagnostic biomarker for CRC.^21^ *MiR‐92a* has ever been reported to be involved in the development and metastasis of CRC and serve as an effective biomarker for the cancer diagnosis and prognosis.^22^, ^23^, ^24^ Therefore, we speculated that the expression of *miR‐92a‐1* might also act as an indicator in early detection of CRC.

In this study, we sought to investigate the expression level of *miR‐92a‐1* in CRC patients, and its association with clinical characteristics. In addition, we explored the diagnostic value of *miR‐92a‐1* in CRC.

## 2 METHODS AND MATERIALS

### 2.1 Patients and sample collection

The present study was a retrospective investigation. A total of 148 CRC patients were recruited from Cangzhou Central Hospital. Inclusion criteria for CRC patients were as follows: (a) pathophysiologically confirmed; (b) not receiving any anti‐tumour treatments previously; (c) possessing available clinical records. Meeting any one of the following conditions, patients would be excluded: (a) without pathological diagnosis; (b) dying within one month after diagnosis; (c) showing abnormal liver/kidney function and routine blood test results or other associated or co‐existing diseases; (d) with other primary malignancies. In addition, 68 gender‐ and age‐matched healthy volunteers were recruited as healthy controls in this study, who experienced physical examination in the physical examination centre of the hospital. None of the healthy individuals had the history of malignancies or abnormalities in liver/kidney function.

Blood samples were collected from CRC patients and healthy volunteers in the morning after fasting for 8‐10h. The blood samples were centrifuged to isolate serum samples and stored at −80℃ for further studies. Meanwhile, we recorded clinicopathological characteristics of the CRC patients, including age, gender, tumour size, tumour location, TNM stage, histological stage, lymph node metastasis and distant metastasis (Table 1). This study was approved by the ethics committee of the hospital. All participants provided written informed consents for this research.

**TABLE 1:** Association of *miR‐92a‐1* with the clinicopathological features of CRC patients

Features	Total N = 148	*MiR‐92a‐1* expression		*P*‐values
		Low (n = 62)	High (n = 86)	
Age (years)				.764
≤60	57	23	34	
>60	91	39	52	
Gender				.721
Male	67	27	40	
Female	81	35	46	
Tumour size (cm)				.471
≤5	76	34	42	
>5	72	28	44	
Location				.785
Colon	64	26	38	
Rectum	84	36	48	
TNM stage				.020
I‐II	74	38	36	
III‐IV	74	24	50	
Histological stage				.003
Well; Moderate	74	40	34	
Poor	74	22	52	
LN metastasis				.003
Yes	72	39	33	
No	76	23	53	
Distant metastasis				<.001
Yes	78	22	56	
No	70	40	30	

### 2.2 RNA extraction and quantitative real‐time polymerase chain reaction (qRT‐PCR)

Total RNAs were extracted from serum samples adopting TRIzol reagent kit (catalogue number: 15596026, Invitrogen) as per the manufacturer's protocols. The ratio of OD260/OD280 (1.9‐2.0) was used to evaluate the purity of isolated RNAs. Total RNA was used for reverse transcription reaction employing TaqMan miRNA reverse transcription kit (catalogue number: 4366596, Applied Biosystems). Obtained cDNA was stored at −20°C until use.

Then, PCR was carried out in ABI 7300 Real‐Time PCR System (Applied Biosystems) with SYBR Green PCR Master Mix (Applied Biosystems). Cycle threshold (CT) was defined as the cycle number of fluorescence signal reaching to threshold. We set threshold level at 0.1‐0.3, and CT values of miRNA samples were automatically calculated based on miRNA abundance.^25^ *U6* gene served as endogenous control to normalize relative expression level of *miR‐92a‐1*. All data were calculated with the method of 2^−ΔΔCt^. Primer sequences were designed based on published data, as follows: *miR‐92a‐1*, forward‐5'‐ACACAGGTTGGGATCGGTTG‐3', and reverse‐5'‐CAAACTCAACAGGCCGGGA‐3'; *U6*, forward‐5'‐CTCGCTTCGGCAGCACA‐3', and reverse‐5'‐AACGCTTCACGAATTTGCGT‐3'.

### 2.3 Statistical analysis

Continuous data were present as mean ± SD and compared adopting Student's *t* test. Chi‐square test was used to examine the correlation of *miR‐92a‐1* with clinicopathological features of CRC patients. Receiver operating characteristic (ROC) analysis was used to evaluate the diagnostic value of *miR‐92a‐1* in CRC. All data analyses were performed in SPSS 21.0 statistical software. *P*‐value less than .05 was considered as significant level.

## 3 RESULTS

### 3.1 Expression level of *miR‐92a‐1*

**FIGURE 1:**
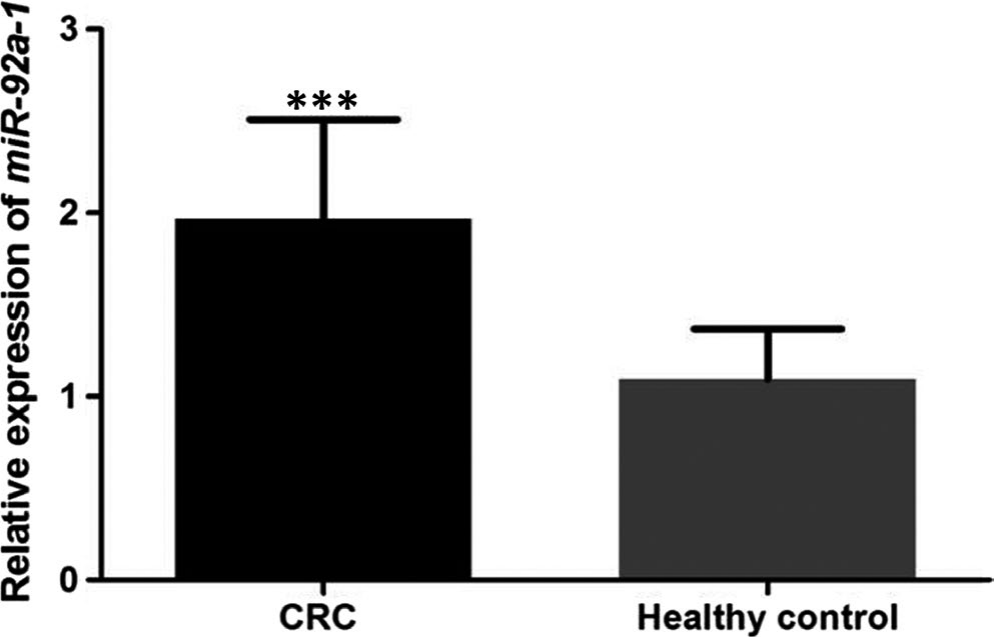
Serum level of *miR‐92a‐1* in CRC patients and healthy individuals. The expression of *miR‐92a‐1* was significantly up‐regulated in patients with CRC compared with the healthy individuals. ***: suggested *P* < .001

According to qRT‐PCR, the expression of *miR‐92a‐1* was significantly increased in CRC patients, compared with healthy controls (Figure 1).

### 3.2 Association of *miR‐92a‐1* with clinicopathological characteristics of CRC

In the current study, CRC patients were divided into high (n = 86) and low (n = 62) expression groups, according to their median expression of *miR‐92a‐1* in CRC tissues. Chi‐square test was used to analyse the relationship between *miR‐92a‐1* expression and clinicopathological profiles of CRC. Analysis results indicated that the overexpression of *miR‐92a‐1* was correlated with advanced TNM stage (*P* = .02), poor histological stage (*P* = .003), positive lymph node metastasis (*P* = .003) and distant metastasis (*P* < .001). There was no significant relationship between *miR‐92a‐1* and age, gender, or tumour size or location (all *P > *.05, Table 1).

### 3.3 Diagnostic value of *miR‐92a‐1* in CRC

**FIGURE 2:**
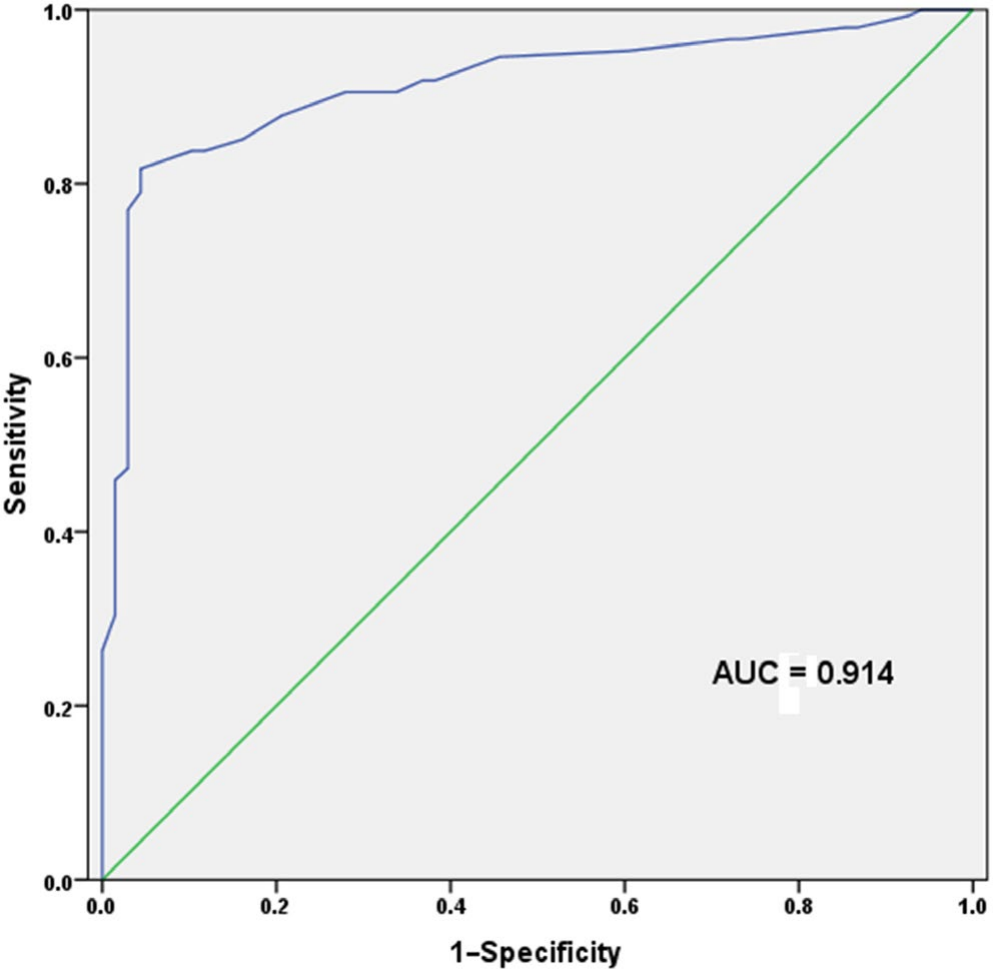
ROC curve constructed based on the expression levels of *miR‐92a‐1* in CRC patients and healthy individuals. Analysis results demonstrated that *miR‐92a‐1* could discriminate between CRC patients and healthy individuals, with the AUC of 0.914, combing with the sensitivity of 81.8% and the specificity of 95.6%. The cut‐off value of *miR‐92a‐1* for CRC diagnosis was 1.485

To explore diagnostic value of *miR‐92a‐1* in CRC, ROC analysis was performed and showed an area under the ROC curve (AUC) of 0.914, suggesting its high diagnostic accuracy in CRC. The cut‐off value of *miR‐92a‐1* for CRC diagnosis was 1.485, with a sensitivity of 81.8% and a specificity of 95.6% (Figure 2).

## 4 DISCUSSION

CRC is a prevalent malignant tumour in the world.^26^ Despite significant improvements in surgical, neoadjuvant chemotherapy and radiotherapy, the survival rates of patients with CRC are still dismal.^27^, ^28^ Early diagnosis is essential for survival in CRC patients.^29^ Until now, the gold standard for CRC diagnosis is colonoscopy, but its invasive nature limits its wide application in clinical practices.^30^ Commonly used serum biomarkers for CRC diagnosis include CEA and CA19‐9. However, both of them only show low accuracy in CRC diagnosis.^31^ Consequently, novel biomarkers with high sensitivity and specificity are urgently required for CRC diagnosis.

MiRNAs, short non‐coding RNAs, play important roles in physiological and pathological conditions. Their expression profiles show close association with tumour development, progression and treatment response, suggesting their indicative functions in human malignancy.^32^ In addition, the expressions of miRNAs are stable and can be easily detected in archived tissue specimens and body fluids.^33^ According to existing documents, miRNAs may provide promising approaches for cancer diagnosis. In previous studies, several miRNAs have been confirmed to play predictive roles in the processes of CRC. Wang et al reported that circulating *miR‐210* level was significantly different between CRC patients and healthy individuals, which showed diagnostic and prognostic capability for the cancer.^34^ *MiR‐223*, as another example, was reportedly up‐regulated in CRC tissues, and its elevated expression was correlated with positive metastasis and poor prognosis in the patients.^35^ In a word, abnormally expressed miRNAs are involved in the aetiology of CRC and might serve as indicators for the cancer.

As a member of *miR‐17‐92* cluster, *miR‐92a* was correlated with the progression of CRC.^22^, ^23^, ^24^ Therefore, we speculated that *miR‐92a‐1*, the precursor of *miR‐92a*, might play a crucial role in the progression of CRC. In the current study, we investigated serum level of *miR‐92a‐1* in CRC patients and healthy individuals via qRT‐PCR. The results suggested that serum *miR‐92a‐1* was up‐regulated in CRC patients compared to healthy individuals. Moreover, increased expression of *miR‐92a‐1* was closely linked to advanced TNM stage, poor histological stage and positive lymph node metastasis and distant metastasis, which suggested that *miR‐92a‐1* might be involved in CRC development and progression. Alterations in *miR‐92a‐1* expression among CRC patients were normal, in comparison with healthy controls, and in further study, we will check the role of *miR‐92a‐1* in the progression of CRC. Experimental data revealed that *miR‐92a‐1*, an oncogene, could promote aggressive progression of CRC. However, specific mechanisms for *miR‐92a‐1* affecting CRC were not investigated in the present study. Further analyses are still required.

The members of *miR‐92a* family play significant roles in the development and progression of CRC and might serve as biomarkers for the cancer.^36^ However, diagnostic performance of *miR‐92a‐1* in CRC remained unclear. To examine diagnostic value of *miR‐92a‐1*, ROC curve was constructed in the current study. The results demonstrated that *miR‐92a‐1* could distinguish CRC patients from healthy individuals with high sensitivity and specificity. *MiR‐92a‐1* might be a potential diagnostic biomarker for CRC. It was worth noting that the sample size in the current study was relatively small, and the application value of *miR‐92a‐1* in CRC diagnosis requires further identifications. Future studies should be performed to verify our findings and investigate relevant mechanism both in vivo and in vitro.

In summary, *miR‐92a‐1* is up‐regulated in CRC patients and correlated with malignant tumour development and progression. Serum *miR‐92a‐1* may be a novel diagnostic biomarker for CRC.

## CONFLICT OF INTEREST

None.

## AUTHOR CONTRIBUTIONS

YS conceived and designed the experiments; ZL conceived and performed the experiments; and YS prepared figures. YS and ZL wrote the main manuscript text. All authors reviewed the manuscript. With the approval of Cangzhou Central Hospital Ethics Committee, written informed consent was obtained from every patient.

## Data Availability

All data generated or analysed during this study are included in this article.
